# Dafachronic acid promotes larval development in *Haemonchus contortus* by modulating dauer signalling and lipid metabolism

**DOI:** 10.1371/journal.ppat.1007960

**Published:** 2019-07-23

**Authors:** Guangxu Ma, Tao Wang, Pasi K. Korhonen, Neil D. Young, Shuai Nie, Ching-Seng Ang, Nicholas A. Williamson, Gavin E. Reid, Robin B. Gasser

**Affiliations:** 1 Department of Veterinary Biosciences, Melbourne Veterinary School, The University of Melbourne, Parkville, Victoria, Australia; 2 Bio21 Mass Spectrometry and Proteomics Facility, The University of Melbourne, Parkville, Victoria, Australia; 3 School of Chemistry, The University of Melbourne, Parkville, Victoria, Australia; 4 Department of Biochemistry and Molecular Biology, The University of Melbourne, Parkville, Victoria, Australia; 5 Bio21 Molecular Science and Biotechnology Institute, The University of Melbourne, Parkville, Victoria, Australia; Max Planck Institute for Developmental Biology, GERMANY

## Abstract

Here, we discovered an endogenous dafachronic acid (DA) in the socioeconomically important parasitic nematode *Haemonchus contortus*. We demonstrate that DA promotes larval exsheathment and development in this nematode via a relatively conserved nuclear hormone receptor (DAF-12). This stimulatory effect is dose- and time-dependent, and relates to a modulation of dauer-like signalling, and glycerolipid and glycerophospholipid metabolism, likely via a negative feedback loop. Specific chemical inhibition of DAF-9 (cytochrome P450) was shown to significantly reduce the amount of endogenous DA in *H*. *contortus*; compromise both larval exsheathment and development in vitro; and modulate lipid metabolism. Taken together, this evidence shows that DA plays a key functional role in the developmental transition from the free-living to the parasitic stage of *H*. *contortus* by modulating the dauer-like signalling pathway and lipid metabolism. Understanding the intricacies of the DA-DAF-12 system and associated networks in *H*. *contortus* and related parasitic nematodes could pave the way to new, nematode-specific treatments.

## Author summary

In the present study, using an integrative multi-omics approach, we show that dafachronic acid (DA) plays a critical functional role in the developmental transition in larvae of the parasitic nematode *Haemonchus contortus* (barber’s pole worm) by modulating the dauer-like signalling pathway and lipid metabolism. The DA-DAF-12 signalling module in *H*. *contortus* provides a paradigm to explore its developmental and reproductive biology at the molecular level, to study physiochemical cross-talk between the parasite and its hosts, and to discover novel anthelmintic targets.

## Introduction

Dafachronic acids (DAs) are bile acid-like, steroidal hormones, which were first discovered in the free-living nematode *Caenorhabditis elegans* [1]. In this nematode, Δ7-DA binds to the nuclear hormone receptor DAF-12 to modulate developmental and reproductive processes in response to changing environmental conditions [2–8]. For instance, under favourable conditions, the DA-DAF-12 module is activated to promote continuous larval development to the adult stage, whereas under unfavourable conditions, this module is inactivated to suppress development, leading to larval arrest (called dauer formation or diapause) [9,10]. This DA-DAF-12 system—essentially a “developmental switch”—is regulated by the dauer signalling pathway, which comprises elements of the cyclic guanosine monophosphate (cGMP), DAF-7 transforming growth factor-β (TGF-β), DAF-2 insulin/insulin-like growth factor 1 (IGF-1) and steroid-hormone signalling cascades [9–11].

The DA-DAF-12 system is not unique to *C*. *elegans*. It has been shown to be functional in the free-living nematode *Pristionchus pacificus* [12] and in the parasitic nematodes of *Ancylostoma ceylanicum* (clade V) and *Strongyloides stercoralis* (clade IV) [13–16]. Using informatic approaches, components of this system have been identified in parasitic nematodes representing different evolutionary clades, including *Trichinella spiralis*, *Trichuris trichiura* (clade I); *Brugia malayi* and *Loa loa* (clade III), [17]; and, recently, DA was discovered in *Ascaris suum* and *Toxocara canis* (ascaridoids; clade III) [18]. Published information indicates that this endocrine system (controlling dauer formation, or developmental arrest) is relatively conserved for members of the phylum Nematoda [19–21], raising interest in the proposition that DAF-12 and/or associated molecules might represent suitable targets for new anthelmintics [13,22–24]. This aspect is particularly important, given the nature and extent of anthelmintic resistance in socioeconomically important parasitic nematodes of animals, and the adverse impact that it has on the agricultural and associated industries through reduced animal productivity [25]. However, surprisingly, as yet there has been no detailed structural or functional investigation of DA-DAF-12 and associated signalling pathways in economically significant nematodes of livestock animals.

The barber’s pole worm, *Haemonchus contortus* (order Strongylida), is particularly well-suited for molecular explorations [26]. It is arguably the most pathogenic nematode of ruminants, and the disease that it causes (haemonchosis) has a major, adverse impact on animal health and production worldwide [27,28]. This worm has a short life-cycle (~ 28–30 days), has major reproductive potential and, thus, can be readily produced in large numbers in experimental sheep, allowing detailed in vitro studies. The worm develops from the egg to the adult stage through four larval stages, with a dauer-like developmental arrest at the third stage (L3) in the environment, and a possible developmental arrest (hypobiosis) at the fourth stage (L4) within the host animal [29–31]. Recently, we established an efficient in vitro-culture system for larval stages of this parasitic nematode [32], which facilitates in-depth studies of developmental processes and mechanisms [33–35], underpinned by extensive genomic resources [36–38] and enabled by a ready accessibility to transcriptomic, proteomic, lipidomic and informatic technologies [33,35,39,40]. Using these resources and technologies, in the present study, we elucidate the functionality of DA-DAF-12 system and explore how it modulates associated pathways in this highly significant parasitic nematode—*H*. *contortus*.

## Results

### Transcriptional changes link to the dauer-signalling pathway during larval transition, and the identification and quantitation of endogenous Δ7-DA

*Haemonchus contortus* undergoes a morphological transition from an infective L3, via the exsheathed L3 (called xL3), to the parasitic L4 stage [29], which can be carried out in vitro [32]. Here, we investigated, the transcription of genes inferred to be linked to dauer-signalling [34] during this transition in vitro, and then identified and quantitated Δ7-DA in respective larval stages of the nematode.

**Fig 1:**
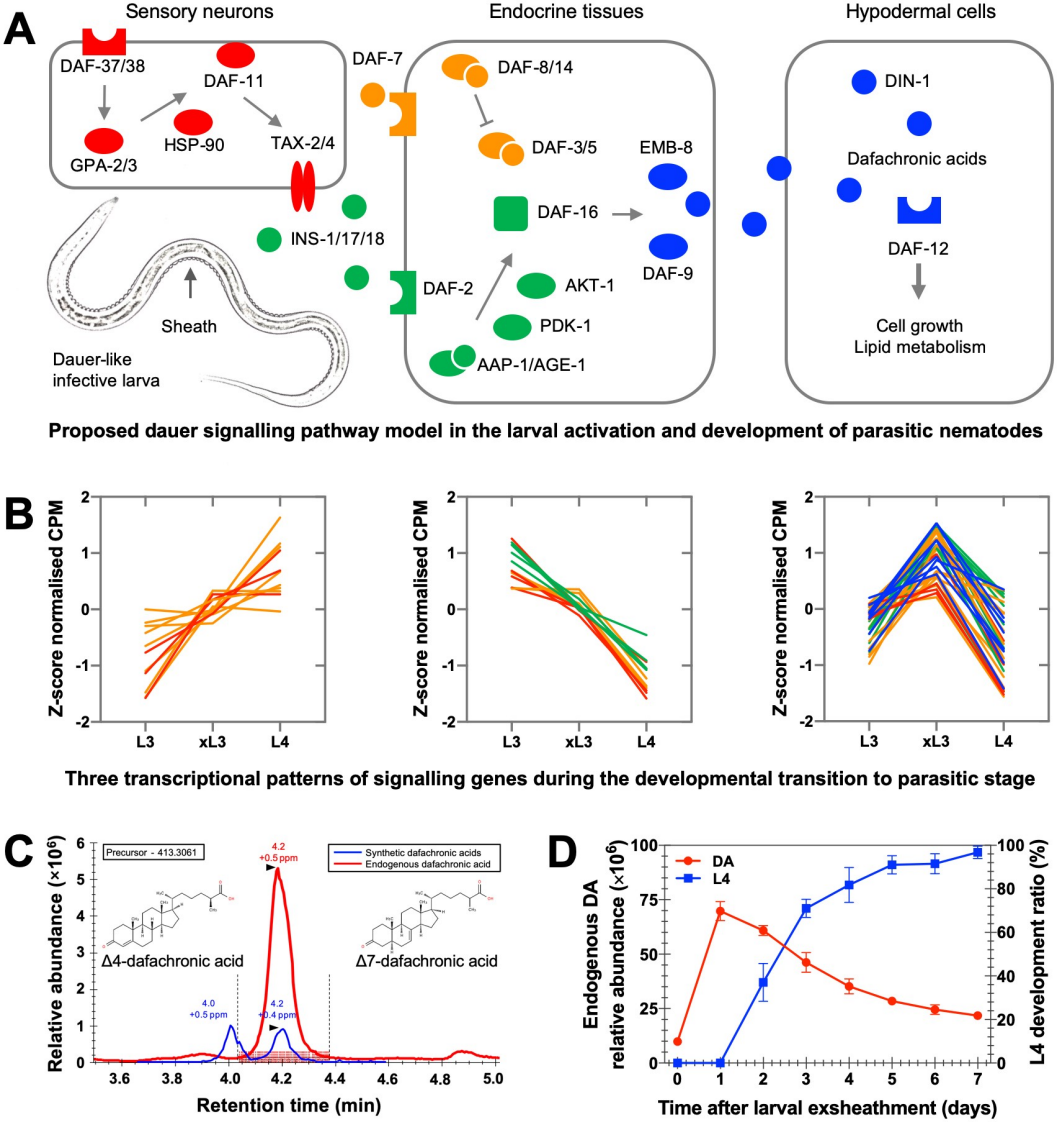
Transcriptional changes pertaining to dauer signalling genes, and quantification of dafachronic acids in *Haemonchus contortus* during developmental transition. (**A**) Model of the cyclic guanosine monophosphate (cGMP) (red), DAF-7-related transforming growth factor-β (TGF-β) (orange), DAF-2-related insulin-like growth factor 1 (IGF-1) (green) and steroid hormone signalling (blue) pathways proposed for *H*. *contortus* [34]. This model is predicted to play a role in integrating environmental signals to control the biosynthesis of one or more dafachronic acids (DAs), which activate the nuclear hormone receptor DAF-12. The DA-DAF-12 module might serve as a checkpoint for developmental decisions and associate with nutrient metabolism in parasitic nematodes [8,14,17,34]. (**B**) Transcriptional profiles (Z-score normalised, mapped reads per million) of 61 gene homologues involved in the cGMP (red), TGF-β (orange), IGF-1 (green) and steroid hormone (blue) signalling pathways are displayed for the developmental transition from the dauer-like third larval stage (L3), via exsheathed L3 (xL3), to the parasitic fourth larval stage (L4) of *H*. *contortus* in vitro. (**C**) Using (25S)-Δ4-DA (calculated mass: 413.3061, retention time: 4.0 min) and (25S)-Δ7-DA (calculated mass: 413.3061; retention time: 4.2 min) as references (blue peaks), endogenous Δ7-DA (retention time: 4.2 min; red peak) was detected in *H*. *contortus* with mass error estimated at 0.5 part per million (ppm). (**D**) The relative abundance of endogenous Δ7-DA following larval exsheathment and in the ensuing larval development in vitro is indicated.

We recorded significant alterations in the transcription of 14 of 61 genes inferred to be involved in dauer signalling in *H*. *contortus* (Fig 1A and 1B): three genes (*Hc-daf-21* [or *hsp-90*], *-scd-1* and *-hsb-1*) were highly upregulated in xL3s *versus* L3s, one (*Hc-daf-36*) was highly upregulated in L4s *versus* xL3s, and 10 genes (*Hc-gpa-2*, -*gpa-3*, *-daf-7*, *-daf-3*, *-scd-2*, *-akt-1*, *-daf-16*, *-emb-8*, *-daf-12* and *-ugt-65*) were substantially downregulated in L4s *versus* xL3s (FC > 2, *P* < 0.01; S1 Table).

Based on prior knowledge for *C*. *elegans* [9], these transcriptional alterations (Fig 1B) suggested that DAs are integral to this developmental transition in *H*. *contortus*, because the biosynthesis of DAs likely represents the outcome of the dauer signalling pathway (Fig 1A) [34]. Therefore, we investigated *H*. *contortus* L3s for the presence of DA. Endogenous Δ7-DA (retention time: 4.2 min; mass error: ~ 0.5 part per million) was unequivocally identified in L3s (Fig 1C), and then quantified in all larval stages studied here (Fig 1D). The abundance of Δ7-DA increased substantially from L3 to xL3 (24 h following exsheathment) and then decreased gradually in the ensuing 6 days of in vitro-culture (Fig 1D).

### Synthetic, exogenous (25S)-Δ7-DA activates *Hc*-DAF-12

**Fig 2:**
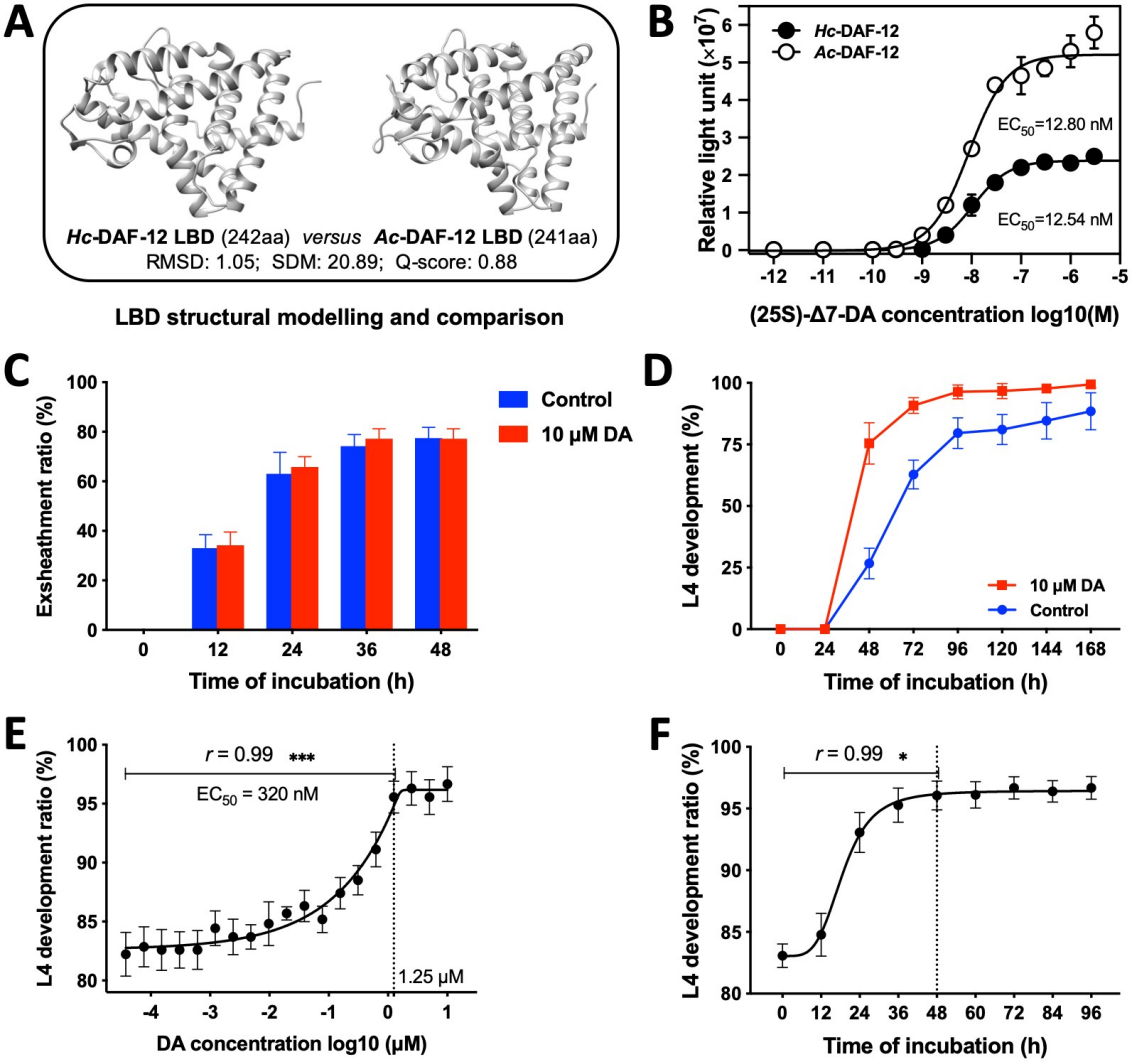
The influence of (25S)-Δ7-DA on larval activation and development. (**A**) Comparison of the ligand-binding domain (LBD) of DAF-12 of *Haemonchus contortus* (*Hc*-DAF-12) with that of *Ac*-DAF-12 from *Ancylostoma caninum*, using the following parameters: sequence length, root-mean-square deviation (RMSD), structural distance measure (SDM) and Q-score. (**B**) Activation of *Hc*-DAF-12 and *Ac*-DAF-12 by (25S)-Δ7-DA) in a luciferase reporter assay. The effects of 10 μM of (25S)-Δ7-DA on (**C**) larval exsheathment and (**D**) larval development. The effect of (25S)-Δ7-DA on larval development is both (**E**) dose- and (**F**) time- dependent. An error bar indicates a standard deviation (SD; four replicates). Statistical significance is indicated with one or more asterisks (*P < 0.05, **P < 0.01, ***P < 0.001, using Student’s t-test).

To examine whether (25S)-Δ7-DA might bind to the ligand-binding domain (LBD) of *Hc*-DAF-12, we compared the structural model of this predicted LBD with that of *Ac*-DAF-12 from *Ancylostoma caninum* (a canine hookworm which is a related strongylid nematode) (cf. [13]). Using three independent algorithms, we showed high structural similarity, achieving a root-mean-square deviation (RMSD) of 1.05, a structural distance measure (SDM) of 20.89 and a Q-score of 0.88 (Fig 2A), suggesting that *Hc*-DAF-12 and *Ac*-DAF-12 have a similar binding affinity and ability to activate DAF-12. This proposal was confirmed by showing that, in a luciferase reporter assay, (25S)-Δ7-DA at 50 nM to 1 μM activated *Hc*-DAF-12 with an EC_50_ of 12.54 nM, which is similar to that of *Ac*-DAF-12 (12.80 nM) (Fig 2B).

### Exogenous (25S)-Δ7-DA stimulates larval growth and development

Knowing that (25S)-Δ7-DA activates *Hc*-DAF-12, we then explored whether we could influence larval exsheathment, growth and/or development in vitro using exogenous, synthetic (25S)-Δ7-DA. There was no significant difference (*P* > 0.05) in exsheathment between L3s and L3s exposed for 48 h to 10 μM of (25S)-Δ7-DA (Fig 2C). However, when cultured for 48 h in the presence of 10 μM of (25S)-Δ7-DA, 49% more xL3s developed to L4s in vitro, and the development from xL3s to L4s was significantly more rapid than unexposed controls (7 days) (Fig 2D). This stimulatory effect of (25S)-Δ7-DA on larval growth and development was both dose- and time-dependent (Fig 2E and 2F). There was a positive correlation between the concentration (*r* = 0.99, *P* < 0.001) of (25S)-Δ7-DA (0–1.25 μM) and larval development (82–96%) after 2 days of culture, and (25S)-Δ7-DA of 1.25, 2.50, 5.00 and 10.00 μM achieved a similar development rate (Fig 2E). Under the condition of treatment with 1.25 μM of (25S)-Δ7-DA, there was a positive correlation (*r* = 0.99, *P* < 0.05) between the time of treatment (0–48 h) and larval development (83–96%) (Fig 2F). The half maximum effective concentration (EC_50_) of (25S)-Δ7-DA on larval development was estimated at 320 nM.

### Establishment of transcriptomic, proteomic and lipidomic data sets for subsequent analyses

To explore molecular responses in *H*. *contortus* associated with DA, we established transcriptomic, proteomic and lipidomic resources. Individual transcriptomic, proteomic and lipidomic data sets were produced for xL3s (0 h), xL3s (24 h) and (25S)-Δ7-DA-treated xL3s (24 h) (four replicates each) (S1A Fig). The ‘larval’ transcriptome, proteome and lipidome comprised 12,217 mRNAs, 1,425 protein groups and 653 lipids (representing 23 classes), respectively; proteins were detected for 10% of all transcripts identified. Principal component analyses showed that the three data sets clustered into three distinct groups (xL3s (0 h), xL3s (24 h) and (25S)-Δ7-DA-treated xL3s (24 h)), and hierarchical cluster analyses indicated differences in mRNA transcription, protein expression and lipid abundance between or among the groups (S1B and S1C Fig).

### Analysis of differential transcription, protein expression and lipid abundance

**Fig 3:**
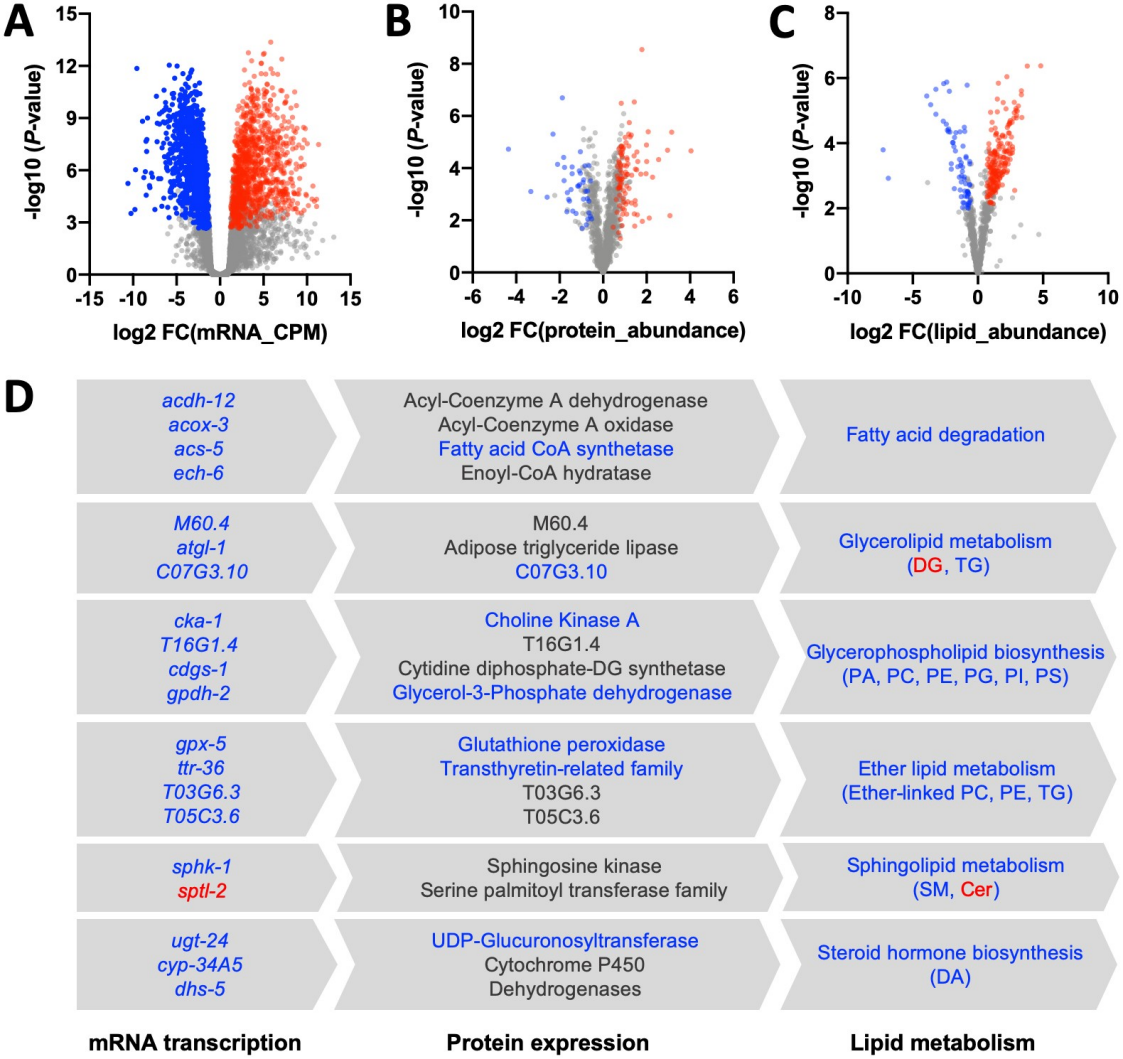
Alterations in mRNA transcription, protein expression or lipid metabolism in *Haemonchus contortus* following larval exsheathment. Differential analyses of (**A**) mRNA, (**B**) protein or (**C**) lipid levels between exsheathed L3s (xL3s) at 0 h and xL3s at 24 h. Indicated is a significant up-regulation (red) or down-regulation (blue) of mRNA transcription, protein expression or lipid levels in larvae (xL3s) 24 h after exsheathment. (**D**) By integrating all results, we showed that molecules (mRNAs encoded by particular genes, proteins and lipids) with significant differential transcription, expression or abundance were specifically associated with fatty acid degradation, glycerolipid metabolism, glycerophospholipid biosynthesis, ether lipid or sphingolipid metabolism and/or steroid hormone biosynthesis. Down-regulated (blue) or up-regulated (red) molecule or pathway indicated; gene and protein designations derived from *Caenorhabditis elegans* homologues (WormBase).

Using individual transcriptomic, proteomic and lipidomic data sets produced (S2, S3 and S4 Tables), we studied molecular changes in *H*. *contortus* xL3s and xL3s exposed to (25S)-Δ7-DA for 24 h. Extensive changes in mRNA transcription, protein expression and lipid abundance were recorded (S2, S3 and S4 Tables). Specifically, significantly higher levels of 1,055 mRNAs, 101 proteins and 180 lipids, and significantly lower levels of 1,029 mRNAs, 46 proteins and 109 lipids were detected in xL3s (at 24 h) compared with L3s immediately following exsheathment (Fig 3A–3C; S2, S3 and S4 Tables). More differences were seen in xL3s exposed to (25S)-Δ7-DA at 24 h, including significantly increased levels of some mRNAs (n = 1,378), proteins (n = 263) and lipids (n = 177) and significantly decreased levels of other mRNAs (n = 1,362), proteins (n = 126) and lipids (n = 109) (S2, S3 and S4 Tables). Most significant molecular changes detected in xL3s (at 24 h) were identified in (25S)-Δ7-DA-treated xL3s (at 24 h); these changes were inferred to be associated with biological processes including environmental information processing (principally signal transduction), genetic information processing (including folding, sorting and degradation) and lipid metabolism (including fatty acid degradation and steroid hormone biosynthesis) (Fig 3D; S2 Fig). Changes in lipid metabolism related predominantly to sphingolipids (ceramide and sphingomyelin), glycerolipids (DG and TG) and glycerophospholipids (PA, PC, PE, PG, PI and PS) (Fig 3D; S4 Table).

### (25S)-Δ7-DA modulates transcription in genes associated with dauer signalling genes and larval growth

**Fig 4:**
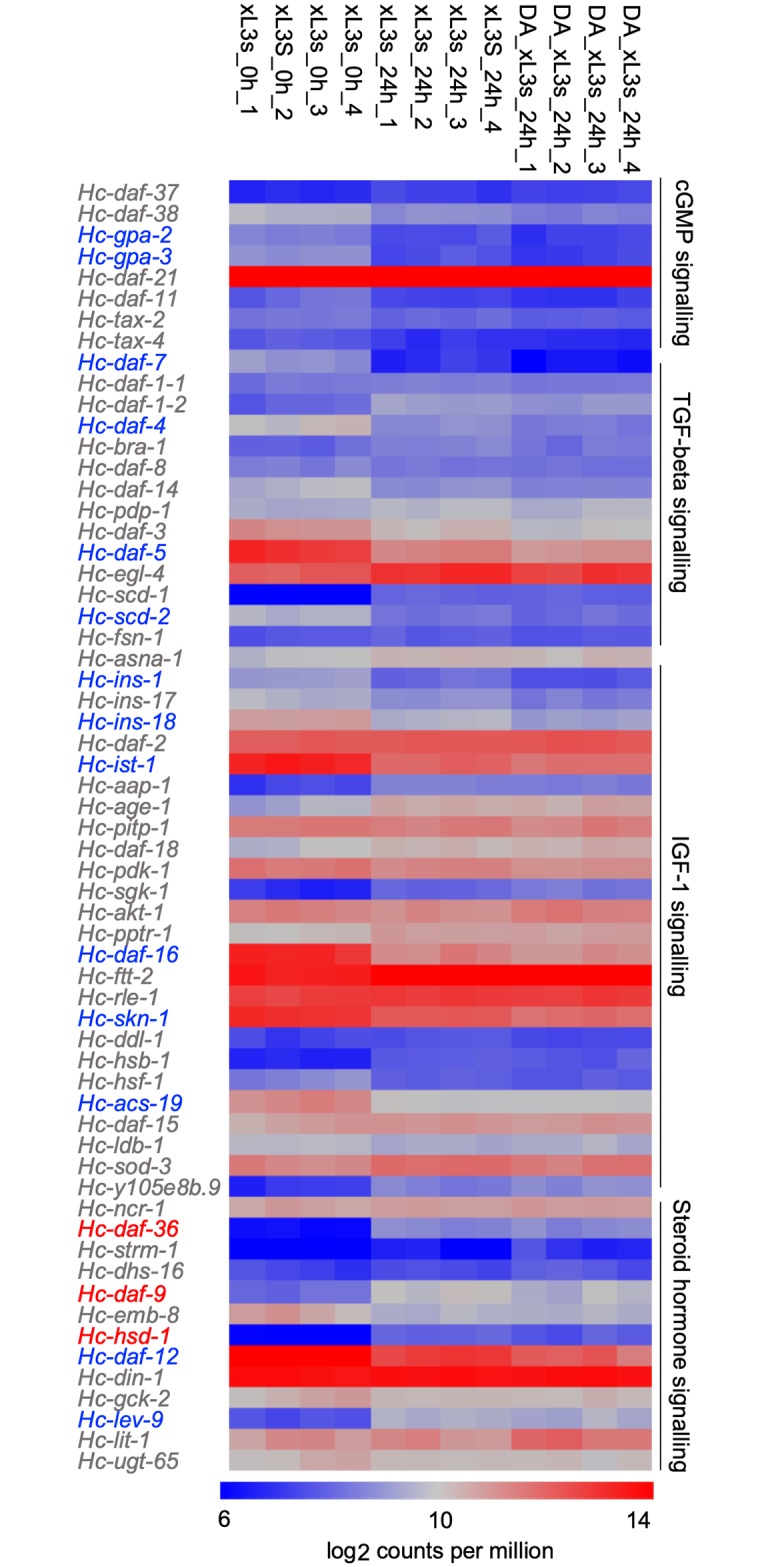
Effect of (25S)-Δ7-DA on the transcription of dauer signalling genes following larval exsheathment. Transcription levels of dauer-like signalling genes in *Haemonchus contortus* exsheathed third-stage larvae (xL3s; 0 h and 24 h) and (25S)-Δ7-DA-treated xL3s (24 h) following exsheathment are indicated in the heat map. Genes involved in the cyclic guanosine monophosphate (cGMP), transforming growth factor-β (TGF-β) and insulin-like growth factor 1 (IGF-1) and steroid hormone signalling pathways are listed. Colour scales indicate scaled read counts per million in the rows; up-regulation (red) or down-regulation (blue) is indicated. Gene designations relate to the dauer-signalling pathway model for *H*. *contortus* [34].

First, we explored differential transcription for genes associated with dauer signalling (Fig 4). In the transition from L3 to xL3s, 24 h following L3 exsheathment, when endogenous DA is at its highest level in xL3s (Fig 1D), transcription in the dauer signalling cascade was downregulated (FC ≥ 2 and *P* < 0.01) for 14 genes involved in cGMP (*Hc-gpa-2* and *-gpa-3*), TGF-β (*Hc-daf-7*, *-daf-4*, *-daf-5* and -*scd-2*), IGF-1 signalling (*Hc-ins-1*, -*ins-18*, -*ist-1*, *-daf-16*, -*skn-1* and -*acs-19*) or steroid hormone signalling (*Hc-emb-8* and *-daf-12*), and transcription was upregulated (FC ≥ 2 and *P* < 0.01) for one gene (*Hc-scd-1*) associated with TGF-β signalling and four genes (*Hc-daf-36*, *-daf-9*, *-hsd-1* and *-lev-9*) linked to steroid hormone signalling (Fig 4; S5 Table). In order to assess whether DA biosynthesis alters the differential transcription of these genes, we undertook an experiment where we added exogenous (25S)-Δ7-DA (1.25 μM) to xL3s for 24 h (after exsheathment). The results showed a further, significant reduction (FC ≥ 2 and *P* < 0.01) in the transcription of 12 downregulated genes (*Hc-gpa-2*, -*gpa-3*, *-daf-7*, *-daf-4*, *-daf-5*, *-scd-2*, *-ins-1*, *-ins-18*, *-ist-1*, *-daf-16*, *-skn-1* and *-daf-12*) and two upregulated genes (*Hc-scd-1* and *-daf-36*) (Fig 4; S5 Table).

**Fig 5:**
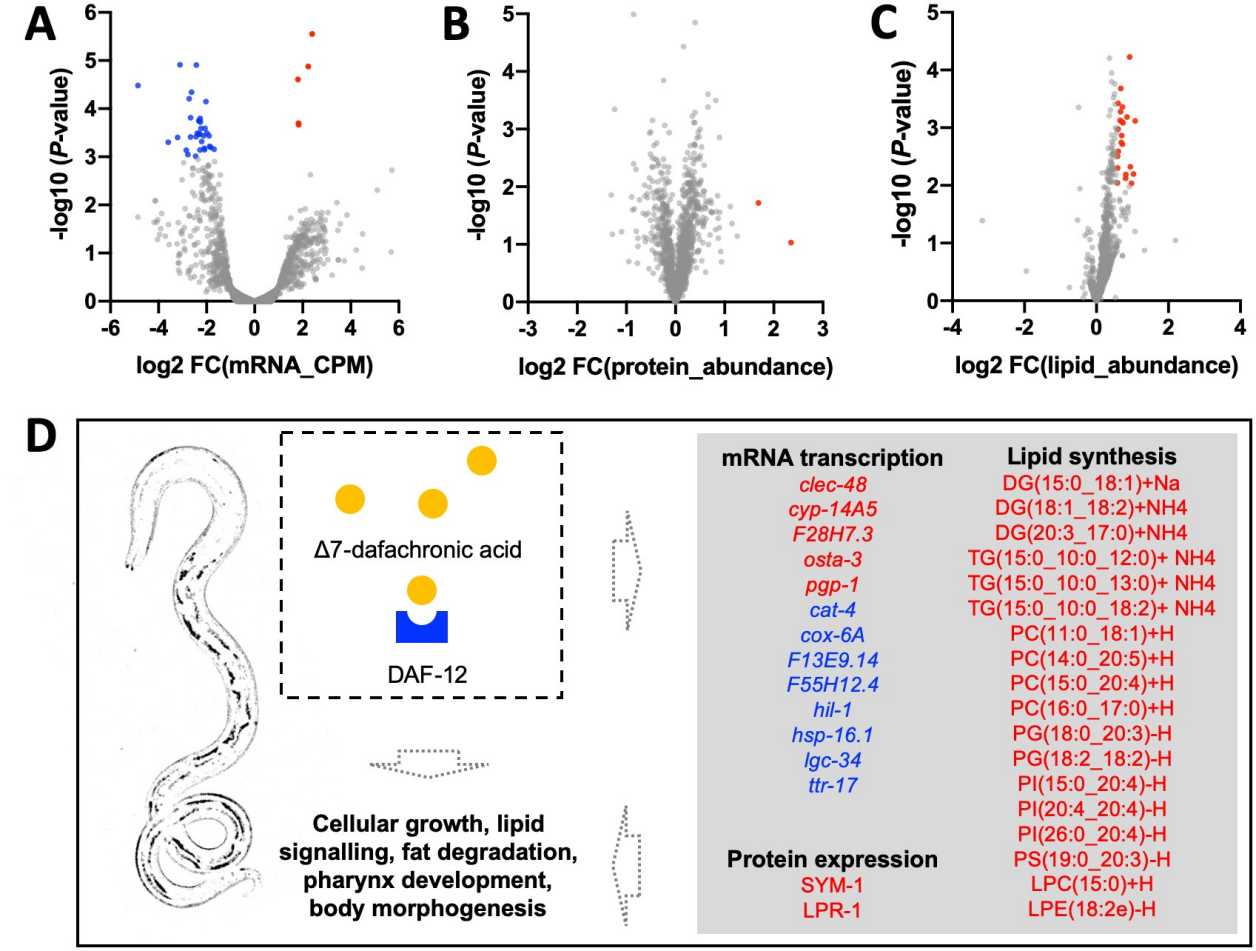
Transcriptomic, proteomic and lipidomic differences between treated and untreated worms. Differential analyses of the (**A**) mRNA, (**B**) protein and (**C**) lipid levels between exsheathed the third-stage larvae (xL3s) and (25S)-Δ7-DA-treated xL3s of *Haemonchus contortus* at 24 h following exsheathment. Molecules that were significantly up-regulated (red) or down-regulated (blue) in (25S)-Δ7-DA-treated xL3s are indicated. (**D**) These molecular alterations inferred to associate with cellular growth, lipid signalling, fat degradation, pharynx development and body morphogenesis; gene and protein designations derived from *Caenorhabditis elegans* homologues (WormBase).

Beyond the dauer signalling pathway, (25S)-Δ7-DA supplementation induced significant (*P* ≤ 0.05) molecular alterations in xL3s following exsheathment. Although a downregulation was recorded exclusively for mRNA transcription of genes *cat-4*, *cox-6A*, *hil-1*, *hsp-16*.*1*, *lgc-34* and *ttr-17*, an upregulation was measured for mRNA transcription (genes *clec-48*, *cyp-14A5*, *osta-3* and *pgp-1*), protein expression (SYM-1 and LPR-3) and lipid abundance [for DG(15:0_18:1), TG(15:0_10:0_18:2), PC(15:0_20:4), PC(16:0_17:0), LPC(15:0) and PI(15:0_20:4)] (Fig 5A–5C; S2, S3 and S4 Tables). These differentially transcribed mRNAs and upregulated proteins were inferred to be involved in larval development, pharynx development and the attachment of body muscle to the extracellular cuticle (body morphogenesis) (Fig 5D; S2 and S3 Tables). The altered abundances of particular glycerolipids and glycerophospholipids were associated with cellular proliferation, lipid signalling and metabolism (Fig 5D).

### Inhibition of endogenous Δ7-DA by dafadine A compromises larval exsheathment and development, and alters lipid abundance

**Fig 6:**
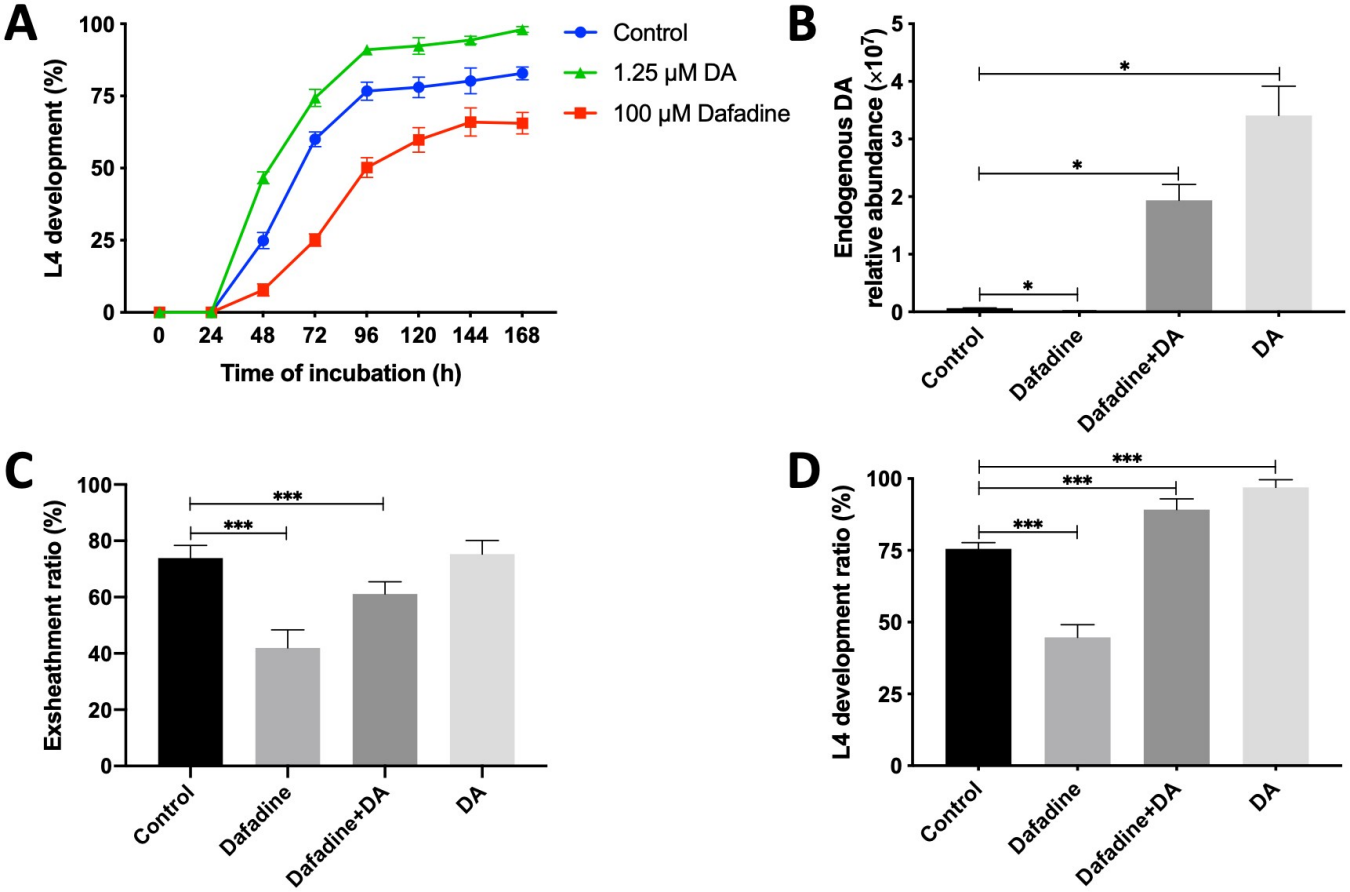
Effects of dafadine A on DA biosynthesis, larval development and lipid metabolism. (**A**) Treatment with 100 μM of dafadine A results in a reduced larval development, which is linked to (**B**) a significantly lower level of endogenous Δ7-DA in dafadine-treated worms. The inhibitory effect of dafadine A and rescuing effect of 1.25 μM of (25S)-Δ7-DA on (**C**) larval exsheathment and (**D**) development. An error bar indicates a standard deviation (SD; four replicates). Statistical significance is indicated with one or more asterisks (*P < 0.05, **P < 0.01, ***P < 0.001, using the Student’s t-test).

As a previous study [41] has shown that dafadine A can specifically inhibit the biosynthesis of DAs in *C*. *elegans*, we elected to test the effect of this inhibitor on Δ7-DA biosynthesis, larval exsheathment and development of *H*. *contortus*. Treatment with dafadine A (100 μM) slowed larval development from the L3 to the L4 stage and significantly reduced the level of endogenous Δ7-DA in dafadine A-treated worms, compared with untreated and (25S)-Δ7-DA-treated worms (Fig 6A and 6B). When L3s were exposed to 100 μM of dafadine A in vitro, exsheathment was significantly inhibited (*P* < 0.001) compared with unexposed L3 controls (Fig 6C). Similarly, when xL3s were exposed to 100 μM of dafadine A, larval development decreased significantly (*P* < 0.001) (Fig 6D). The inhibitory effects of dafadine A on the production of endogenous Δ7-DA, larval exsheathment and development were partially or completely “rescued” by supplementation with 1.25 μM of (25S)-Δ7-DA (*P* < 0.001) (Fig 6B–6D).

**Fig 7:**
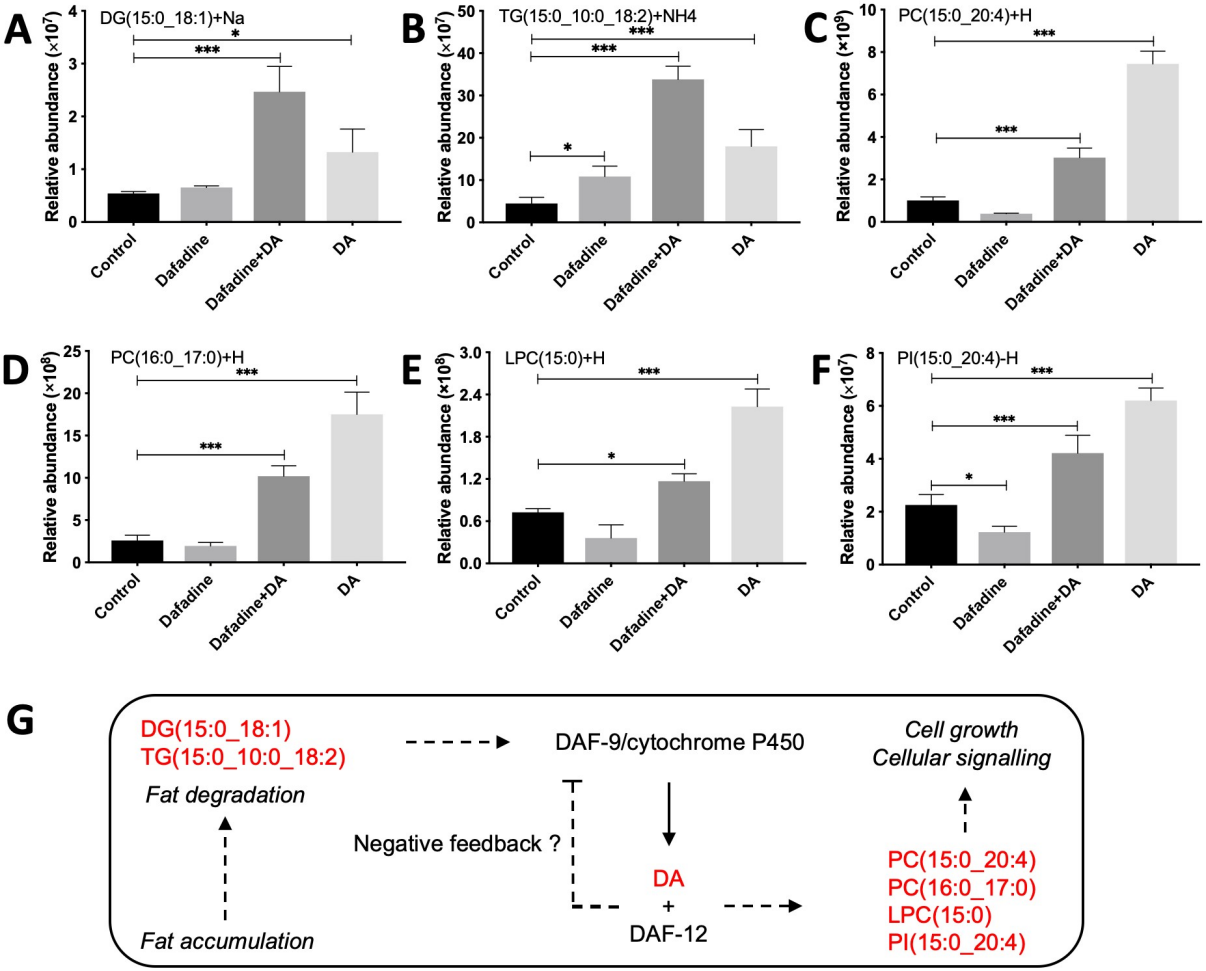
Effects of dafadine A (100 μM) on the abundances of glycerolipids and glycerophospholipids. The abundances of particular (**A**) diradylglycerol (DG), (**B**) triacylglycerol (TG), (**C** and **D**) phosphatidylcholine (PC), (**E**) lysophosphatidylcholine (LPC) and (**F**) phosphatidylinositol (PI) in untreated xL3s; dafadine A-inhibited xL3s; 1.25 μM of (25S)-Δ7-DA-rescued xL3s; and 1.25 μM of (25S)-Δ7-DA-treated xL3s. An error bar indicates a standard deviation (SD; four replicates). Statistical significance is indicated with asterisk (*P < 0.05, **P < 0.01, ***P < 0.001, using the Student’s t-test). (**G**) A schematic showing the DA-DAF-12 module and its proposed functional roles in regulating fat degradation/accumulation, cell growth and cellular signalling. It is proposed that DA produced by DAF-9 (cytochrome P450) activates the nuclear hormone receptor DAF-12, which promotes the degradation of glycerolipids [e.g., DG(15:0_18:1) and TG(15:0_10:10_18:2)] for the subsequent production of glycerophospholipids [e.g., PC(15:0_20:4), PC(16:0_17:0) and PI(15:0_20:4)], and which negatively regulates DA biosynthesis to reduce lipid degradation for fat accumulation. The solid arrow indicates the production of endogenous DA; a dashed line with an arrow indicates an indirect pathway; and a dashed line with a bar indicates a negative feedback loop.

Since the altered abundances of particular glycerolipids and glycerophospholipids were linked to (25S)-Δ7-DA treatment (Fig 5D), we explored the relationship between these molecules and DA in *H*. *contortus*. Treatment with dafadine A (100 μM) altered the abundances of DG(15:0_18:1), TG(15:0_10:0_18:2), PC(15:0_20:4; 16:0_17:0), LPC(15:0) and PI(15:0_20:4) in xL3s at 24 h. Specifically, dafadine A significantly (*P* < 0.01) increased the levels of DG(15:0_18:1) and TG(15:0_10:0_18:2); the levels of these lipids increased further when exposed to (25S)-Δ7-DA (*P* < 0.05) with reference to untreated controls (Fig 7A and 7B). It was also evident that treatment with dafadine A for 24 h significantly reduced levels of PC (15:0_20:4, 16:0_17:0), LPC (15:0) and PI (15:0_20:4) (*P* < 0.05) compared with untreated controls, which were reversed by supplementation with 1.25 μM of (25S)-Δ7-DA (Fig 7C–7F).

## Discussion

This study identified, for the first time, DA in the strongylid nematode *H*. *contortus*, and showed that this hormone promotes larval exsheathment and development via a relatively conserved nuclear hormone receptor, DAF-12. In *H*. *contortus*, the DA-DAF-12 complex modulates the dauer-like signalling pathway, via a negative feedback circuit, and affects molecular alterations linked to pharynx development, body morphogenesis, cellular growth, lipid signalling and metabolism.

The Δ7-DA signal that induces larval development in *H*. *contortus* is transduced via DAF-12. Since the relationship between DA-DAF-12 and developmental regulation in *C*. *elegans* is well-established [4,5,42], a conserved DA-DAF-12 module had been proposed for parasitic nematodes [13,15,21]. Recently, we also showed quite marked sequence and/or structural similarity in the inferred DAF-12 ligand-binding domain (LBD) between *H*. *contortus* and other individual strongylids (*A*. *ceylanicum* and *Necator americanus*) or rhabditids (*S*. *stercoralis* and *C*. *elegans*) [34], suggesting relative functional conservation in dafachronic acid binding and signalling. Indeed, here we confirmed that (25S)-Δ7-DA activates *Hc*-DAF-12 in a luciferase reporter assay, with an EC_50_ (12.54 nM) that is similar to *Ac*-DAF-12 (12.80 nM) from *A*. *caninum*. These findings suggest that the endogenous Δ7-DA signal is transduced by DAF-12 to promote larval development in *H*. *contortus*, consistent with *Ancylostoma* and *Strongyloides* spp. (cf. [12,13,15,16]).

It has been reported that the binding of DA to DAF-12 in parasitic nematodes is similar to that of bile acids to the farnesoid X receptor in mammals, suggesting that a bile acid-like signalling pathway exists in parasitic nematodes [15]. Interestingly, a common hormone-theme has been proposed for physicochemical communications between parasite and host animal [43–45]. A good example of this is that prolactin evokes the transmammary transmission of larvae of the ascaridoid nematode *T*. *canis* in mice [46]. It is readily possible that the DA-DAF-12 module in the latter nematode plays a role in regulating or signalling larval activation and transmission, but this involvement needs to be verified molecularly. Clearly, understanding the functionality of the DA-DAF-12 module in parasitic nematodes could provide a paradigm for exploring cross-talk between parasite and host, particularly for worms which can enter into or exit from hypobiosis (arrested development) in their host, such as some members of the families Trichostrongylidae and Ascarididae [47,48].

In *H*. *contortus*, the upregulated transcription of particular dauer signalling genes during the developmental transition from the L3 (dauer-like) stage to the L4 stage indicates an active DA biosynthesis in xL3s, confirmed by measuring an increase in the level of Δ7-DA following L3 exsheathment. These alterations are similar to the transcriptional changes and hormone signal amplification seen in *C*. *elegans* during its development to the reproductively-active adult stage [6,11]. By contrast, a decreased level of Δ7-DA during the ensuing larval development indicates a reduction of its biosynthesis, which is supported by the observation of a pronounced downregulation of transcription of particular genes linked to dauer-like signalling. The dynamics of these changes in DA and transcription levels suggest that the endogenous synthesis of Δ7-DA is relatively tightly modulated or controlled via an, as yet, uncharacterised feedback circuit. A similar negative feedback mechanism exists in *C*. *elegans*, and operates via the *let*-7 family of microRNAs [5,6]. As *let-7* homologues have not yet been identified or characterised in *H*. *contortus*, further work is required to establish how this feedback mechanism works in this parasitic nematode. Interestingly, Δ4-DA was not detected in *H*. *contortus*, which might be due to its absence or undetectable levels in the larval stages studied. However, both Δ4-DA and Δ7-DA have been detected in both *A*. *suum* and *T*. *canis* at differing levels [18], suggesting a functional distinctiveness of the two isomers in their involvement in selected biological processes in the latter two nematodes.

Different “signal intensity thresholds” of DA might be required for larval activation versus development; we found that 100 μM of (25S)-Δ7-DA did not induce exsheathment (although there is a possibility that DA does not penetrate the L3 sheath), but did significantly stimulate larval development following exsheathment. The specific inhibition of DAF-9 (cytochrome P450) with dafadine A resulted in a significant reduction of both endogenous Δ7-DA levels and larval exsheathment/development, which could be partially reversed through the supplementation of an excess (1.25 μM) of exogenous (25S)-Δ7-DA. These findings are distinct from those described for *Ancylostoma caninum* in that (25S)-Δ7-DA can directly activate infective larvae (L3s) of *A*. *caninum* and can induce post-parasitic larvae of *S*. *stercoralis* to develop to free-living stages [13,16]. The distinct responses to (25S)-Δ7-DA among *H*. *contortus* (clade V), *A*. *caninum* (clade V) and *S*. *stercoralis* (clade IV) might relate to evolutionary divergences in DA-associated signalling pathways within the phylum Nematoda [14,49]. This proposal warrants future evaluation.

Exogenous (25S)-Δ7-DA-induced changes in mRNA, protein and lipid profiles in xL3s of *H*. *contortus* appear to link to phenotypic distinctiveness (development) and lipid metabolism (i.e. fatty acid degradation, and glycero- and glycerophospho-lipid biosynthesis) via the DA-DAF-12 module. To test the functionality of the DA-DAF-12 module, we blocked the biosynthesis of Δ7-DA using a specific inhibitor (i.e. dafadine A) of DAF-9 (cytochrome P450) [41], which resulted in a reduction of the endogenous Δ7-DA level, and, consequently, inhibited larval development. Similar results were achieved when the cytochrome P450s of *Nippostrongylus brasiliensis* and *S*. *stercoralis* were targeted with a less specific inhibitor, ketoconazole [16,50]. The significant reduction of PC(15:0_20:4, 16:0_17:0), LPC(15:0) and PI(15:0_20:4) levels in dafadine A-treated *H*. *contortus* larvae could be reversed by supplementation with (25S)-Δ7-DA, indicating a direct or indirect role for DA-DAF-12 signalling in the metabolism of selected glycerolipids and glycerophospholipids [51]. In addition, the increases in DG(15:0_18:1) and TG(15:0_10:0_18:2) levels in dafadine A-treated worms suggest a role for DAF-9 in glycerolipid metabolism. Interestingly, all of these lipid species are odd-chain fatty acids, which contrasts the situation in *C*. *elegans*, in which only small amounts of straight, odd-chain fatty acids (likely originating from the worm’s food source—*Escherichia coli*) accumulate in lipids [52]. Surprisingly little is known about the origin and functional roles of these odd-chain lipid species in developmental processes of nematodes. Nonetheless, based on the present findings, we propose a dual role for the DA-DAF-12 module in promoting the metabolism of key glycerophospholipids and inhibiting the degradation of some lipids (possibly promoting fat accumulation), which functions via a negative feedback to DAF-9 (see Fig 7G), but, clearly, this hypothesis requires rigorous testing.

Taken together, the findings of the present study provide evidence for a signalling cascade in *H*. *contortus*, in which host signals (e.g., CO_2_, pH, insulin and/or metabolites of bile acids) bind to chemoreceptors, which trigger signal transduction from chemosensory neurons to endocrine cells and then hypodermal cells through the interconnected cGMP, TGF-β and IGF-1 pathways. The transduced signal promotes the metabolism of steroids and the biosynthesis of DA, the latter of which activates the nuclear hormone receptor DAF-12, leading to gene transcription and protein expression associated with body morphogenesis and pharynx development as well as lipid metabolism. A high level of DA would modulate phosphatidylinositol signalling that activates PI3K-AKT signalling [53], resulting in phosphorylation-dependent cytoplasmic sequestration of the transcription factors DAF-16/FOXO [9,54]. The activation of DAF-16/FOXO antagonises the upstream cGMP, TGF-β and IGF-1 signalling [53,55], downregulating DA biosynthesis in a feedback circuit, resulting in a reduced lipid metabolism, and, consequently, in fat accumulation (Fig 7G). Understanding the biosynthesis of DAs and nuclear-hormone signal transduction (e.g., via DA-DAF-12) should provide valuable insights into the developmental biology and adaptation of parasitic nematodes to host animals. Experimental evidence [13,23,24] has already shown that *S*. *stercoralis* hyperinfection can be prevented by treatment with (25S)-Δ7-DA. Although (25S)-Δ7-DA might regulate developmental processes in *H*. *contortus* (order Strongylida) differently from those in *Strongyloides* [12,13,16], the potential of DAF-9 and DAF-12 as novel intervention targets (cf. [13,22]) should be explored further. Clearly, major success achieved in a recent study [56] opens the door to assessing the functional essentiality of these steroid hormone signalling components in *Strongyloides* species by RNA interference.

In conclusion, current findings for *H*. *contortus* indicate that the hormonal signal complex DA-DAF-12 modulates the dauer-like signalling pathway through a feedback loop, and regulates biological processes associated with cellular growth and lipid metabolism via a conserved DA-DAF-12 signalling module during developmental transition. This module provides a paradigm to investigate aspects of the developmental and possibly reproductive biology of *H*. *contortus* and related nematodes, to explore physiochemical cross-talk between parasite and host, and to discover novel intervention strategies against parasitic diseases.

## Methods

### Ethics approval

*Haemonchus contortus* (Haecon-5 strain) was produced in Merino lambs (6 months of age; Victoria, Australia), maintained under helminth-free conditions in facilities in the University of Melbourne. The procedures for animal maintenance and experiments were approved by the University of Melbourne (permit no. 1714374), which follows Part 3 of the Prevention of Cruelty to Animals Act 1986 and Part 4 of the Prevention of Cruelty to Animals Regulations 2008 of the State of Victoria as well as the Australian Code for the Care and Use of Animals for Scientific Purposes (1969).

### *H*. *contortus* stages

A monospecific infection of *H*. *contortus* was maintained in sheep under well-controlled experimental conditions [57]; three distinct larval stages of this nematode were produced in vitro using established methods [32]. In brief, third-stage larvae (L3s) were collected from coproculture, purified and maintained at 10 °C in a refrigerated incubator; exsheathed L3s (xL3s) were produced using a well-established hypochlorite-treatment method [32]; and xL3s were cultured (300 per well of 96-well culture plates), under standardised conditions, in Luria Bertani medium (LB) supplemented with Antibiotic-Antimycotic (cat no. 15240–062, Gibco) (LB*) at 38 °C, 10% v/v CO_2_ to yield fourth-stage larvae (L4s) of *H*. *contortus*.

### Detection of DA in the worm

Endogenous DA was identified by liquid chromatography-mass spectrometric (LC-MS) analysis of lipids extracted from three distinct developmental stages of *H*. *contortus*. Lipids were extracted from four replicates (each 1 mg dry weight) of each L3s, xL3s and L4s using an established method [34]. Each replicate was suspended in ice-cold methanol (40%), homogenised using zirconium oxide beads (ZROB05, Next Advance, USA) and extracted with chloroform:methanol (2:1) by centrifugation at 10,000 ×*g* for 15 min, dried and resuspended in methanol (100%), then subjected to LC-MS analysis in an Orbitrap Fusion Lumos mass spectrometer coupled to an Ultimate 3000 UHPLC using a C30 column (2.1 × 250 mm) (Thermo Fisher Scientific, San Jose, CA, USA). Endogenous DA were identified by comparison with the reference standards (25S)-Δ7-DA and (25S)-Δ4-DA (cat. no. 23017-97-2; Cayman Chemical, USA) (exact mass: 413.3061). Peak areas of extracted ion chromatogram were calculated using Skyline v.4.2.

### Structure modelling and DAF-12 reporter assay

The structure of the LBD of DAF-12 of *H*. *contortus* (*Hc*-DAF-12) (using the inferred amino acid sequence: GenBank accession no. MK_256962) was modelled using the program I-TASSER [58] and compared with that of *A*. *caninum* (*Ac*-DAF-12) [15] using UCSF Chimera v.1.12 [59]. Structural similarities between query and template sequences were established using sequence length, overall RMSD, SDM and Q-score.

To test whether *Hc*-DAF-12 can be activated by (25S)-Δ7-DA, a well-established reporter assay was performed as described previously [1,13]. In brief, HEK293 cells were transfected with the luciferase reporter (50 ng), CMX-β-galactosidase reporter (10 ng) or *Hc*-DAF-12 expression plasmids (15 ng). Ethanol or (25S)-Δ7-DA (0 to 1 μM) was added to cells (8 h following transfection) and incubated for 16 h. Luciferase activity was measured with reference to a CMX-β-galactosidase control.

### Assaying the effect of (25S)-Δ7-DA on larval exsheathment and development

First, L3s (300 worms per well; four replicates) were exsheathed by incubating them at 38 °C and 10% v/v CO_2_ for 48 h in physiological saline in the presence or absence of 10 μM of (25S)-Δ7-DA. The number and percentage of exsheathed L3s (xL3s) were assessed every 12 h. Second, xL3s (300 worms per well; four replicates) were cultured to L4s at 38 °C, 10% v/v CO_2_ in LB* in the absence or presence of (25S)-Δ7-DA (10 μM to 10*2^−17^ μM). The numbers of xL3s and L4s in culture were calculated every 24 h, and the proportion of L4s was calculated at each time point. Statistical analyses (student’s t-test, Spearman’s rank correlation and non-linear regression) were performed using Prism 7 (GraphPad, La Jolla, USA).

### Transcriptomic, proteomic and lipidomic analyses

The transcriptomes, proteomes and lipidomes were produced from *H*. *contortus* xL3s, which had been exsheathed using the established hypochlorite-treatment method [32] and then incubated in LB* (38 °C, 10% v/v CO_2_ for 24 h) in the presence or absence of 1.25 μM (25S)-Δ7-DA. For each treatment, four replicates of 30,000 xL3s each were incubated at 38 °C, 10% v/v CO_2_ for 24 h.

For transcriptomic analysis, total RNA was extracted from each of the replicates of xL3s, processed and sequenced as described previously [33]. In brief, strand-specific mRNA libraries were constructed using the TruSeq RNA Library Prep Kit (Illumina) and sequenced on the BGISEQ-500 platform. Raw reads were processed and mapped to predicted genes of *H*. *contortus* (BioProject: PRJEB506) using Bowtie v.2.1.0 [60] within the software package RSEM v.1.2.11 [61].

For proteomic analysis, proteins were isolated from the replicates as described previously [33]. In brief, protein (50 μg) samples were reduced with Tris(2-carboxyethyl)phosphine (TCEP), alkylated with iodoacetamide and digested with Lys-C/trypsin Mix (cat no. V5072; Promega, USA). The digested samples were acidified with 1.0% (v/v) formic acid and purified using Oasis HLB cartridges (cat no. 186000383; Waters, USA) and then subjected to LC-MS/MS analysis using a QExactive plus Orbitrap mass spectrometer (Thermo Fisher Scientific, USA) with a nanoESI interface in conjunction with an Ultimate 3000 RSLC nanoHPLC (Dionex Ultimate 3000). Mass spectrometry data were analysed using MaxQuant [62] to identify and quantify peptides.

For lipidomic analysis, lipids were extracted from the replicates and analysed by LC-MS/MS using an Orbitrap Fusion Lumos mass spectrometer [40]. For the semi-quantitation of identified lipids, the Splash Lipidomix Mass Spec. Standard (cat no. 330707-1EA, Avanti Polar Lipids, USA), including phosphatidylcholine [PC, 15:0_18:1(d7)], phosphatidylethanolamine [PE, 15:0_18:1(d7)], phosphatidylserine [PS, 15:0_18:1(d7)], phosphatidylglycerol [PG, 15:0_18:1(d7)], phosphatidylinositol [PI, 15:0_18:1(d7)], lysophosphatidylcholine [LPC, 18:1(d7)], lysophosphatidylethanolamine [LPE, 18:1(d7)], monoradylglycerol [MG, 18:1(d7)], diradylglycerol [DG, 15:0_18:1(d7)] and triacylglycerol [TG, 15:0_18:1(d7)_15:0], was used as the internal standard. Additional PS[15:0_18:1(d7)], MG[18:1(d7)] and DG[15:0_18:1(d7)] were supplemented to reach a final concentration of 100 μg/ml for each lipid species. Lipids were identified and quantified using LipidSearch software v.4.2.20 (Thermo Scientific), and manually curated.

The mRNAs, proteins and lipids quantified were subjected to principal component and hierarchical cluster analyses using the Perseus computational platform [63,64]. Differential transcription was explored using the limma, glimma and edgeR packages [65]; a fold-change (FC) of ≥ 2 and a false discovery rate (FDR) of ≤ 0.01 defined a significant difference, unless otherwise stated (FC ≥ 2 and *P* ≤ 0.01). Differential protein expression analysis was conducted using the program Perseus v.1.6.1.1, employing an FC of ≥ 1.5 and an FDR ≤ 0.05 as thresholds. For lipids, an FC of ≥ 1.5 and a *P* value of ≤ 0.01 were used as cut-offs. Differentially transcribed mRNAs and expressed proteins were assigned to Kyoto Encyclopedia of Genes and Genomes (KEGG) Orthology (KO) terms using BlastKOALA [66], and KEGG annotations were analysed and displayed using FuncTree 2 [67].

### Inhibition of DAF-9 (cytochrome P450) in the worm

Dafadine A (cat. no. SML0736; Sigma-Aldrich) is known to specifically inhibit DAF-9 in *C*. *elegans* [41]. This chemical was used to inhibit the endogenous production of DAs. In brief, worms (300 L3s or xL3s per well) were exposed to dafadine A (100 μM) in LB* and incubated at 38 °C, 10% CO_2_ for 7 days. DMSO, (25S)-Δ7-DA (1.25 μM), and dafadine A (100 μM) + (25S)-Δ7-DA (1.25 μM) were used as different controls. DA levels, larval development and lipid abundances were analysed between treated and untreated worms.

### Accession numbers

Nucleic acid sequence data from this study are available via the National Center for Biotechnology Information (NCBI) sequence reads archive (SRA) under accession numbers SUB3797117 and SUB5228712. The proteomic data obtained by mass spectrometry have been deposited in the ProteomeXchange Consortium via the PRIDE partner repository and are linked to the dataset identifier PXD012878.

## Supporting information

S1 Table Transcription profiles of dauer signalling genes during the developmental transition from the free-living L3 stage to the parasitic L4 stage of *Haemonchus contortus* in vitro. (XLSX)

S2 Table Effect of (25S)-Δ7-DA on gene transcription during the developmental (larval) transition of *Haemonchus contortus* in vitro. (XLSX)

S3 Table Effect of (25S)-Δ7-DA on protein expression during the developmental (larval) transition of *Haemonchus contortus* in vitro. (XLSX)

S4 Table Effect of (25S)-Δ7-DA on lipid abundance during the developmental (larval) transition of *Haemonchus contortus* in vitro. (XLSX)

S5 Table Effect of (25S)-Δ7-DA on the upstream dauer signalling pathway during the developmental (larval) transition of *Haemonchus contortus* in vitro. (XLSX)

S1 Fig Transcriptomic, proteomic and lipidomic datasets for the exsheathed third-stage larvae (xL3s) and (25S)-Δ7-DA-treated xL3s. (**A**) Transcriptome, proteome and lipidome produced from xL3s (0 h and 24 h) and xL3s exposed to (25S)-Δ7-DA (24 h). (**B**) Principal component analyses and (**C**) hierarchical clustering of the transcriptomic, proteomic and lipidomic datasets.
(TIF)

S2 Fig Annotation and integration of transcriptomic and proteomic data sets. Functional annotation of mRNAs and proteins differentially transcribed/expressed between exsheathed L3s (xL3s) at 0 h and xL3s at 24 h. Annotation using the Kyoto Encyclopedia of Genes and Genomes (KEGG) database (employing Orthologue (red), Module (orange), Pathway (yellow) and BRITE levels 2 (green) and 1 (blue); see Materials and methods section). Significantly up-regulated (red) or down-regulated (blue) molecules and pathways are indicated.
(TIF)
