# Supplementary figures and images for: Dafachronic acid promotes larval development in Haemonchus contortus by modulating dauer signalling and lipid metabolism
Source: PLoS Pathog. 2019 Jul 23;15(7):e1007960. doi: 10.1371/journal.ppat.1007960 (PMC6677322; doi:10.1371/journal.ppat.1007960)

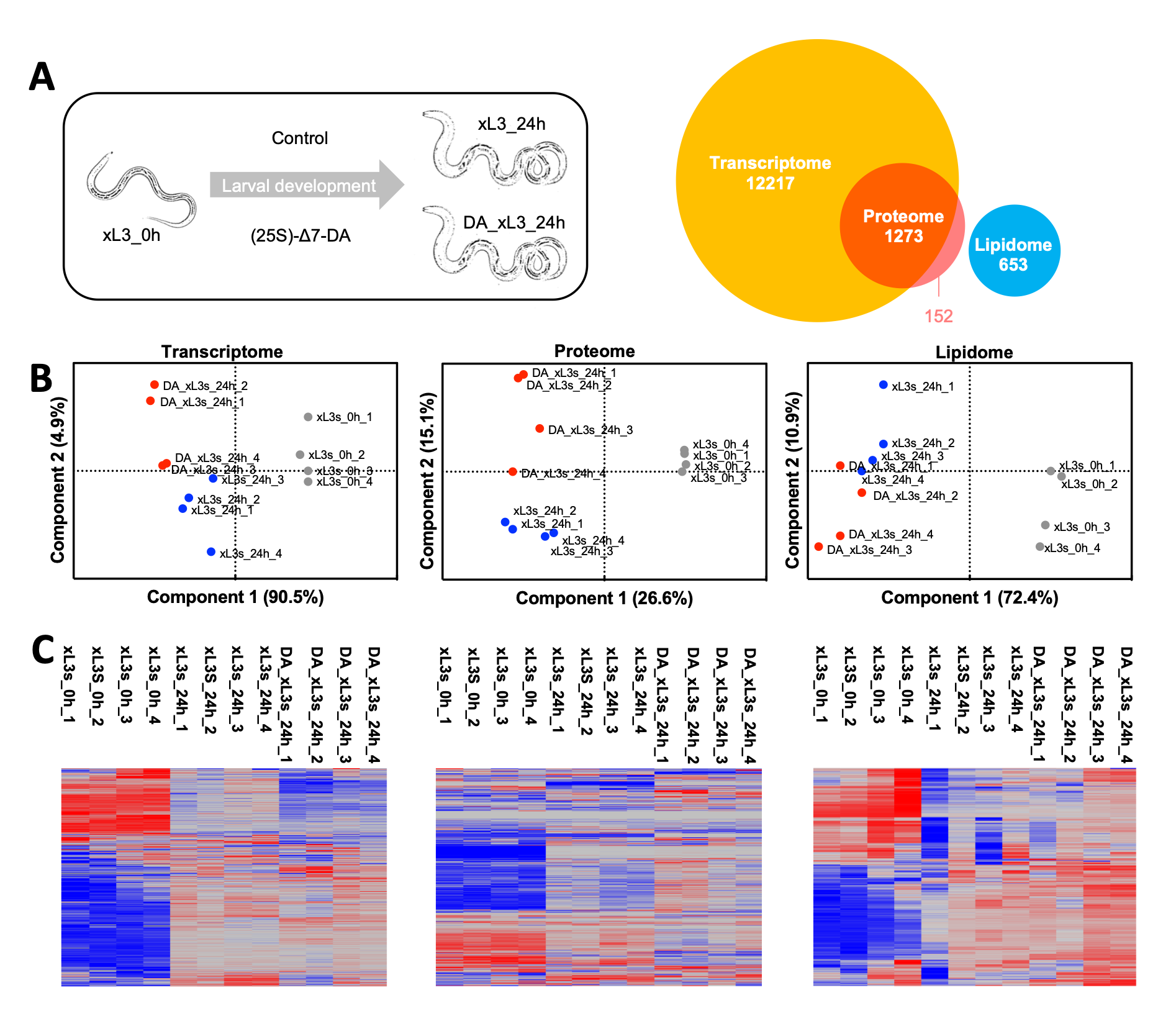

Supplement: S1 Fig — (A) Transcriptome, proteome and lipidome produced from xL3s (0 h and 24 h) and xL3s exposed to (25S)-Δ7-DA (24 h). (B) Principal component analyses and (C) hierarchical clustering of the transcriptomic, proteomic and lipidomic datasets. (TIF) [file ppat.1007960.s006.tif]

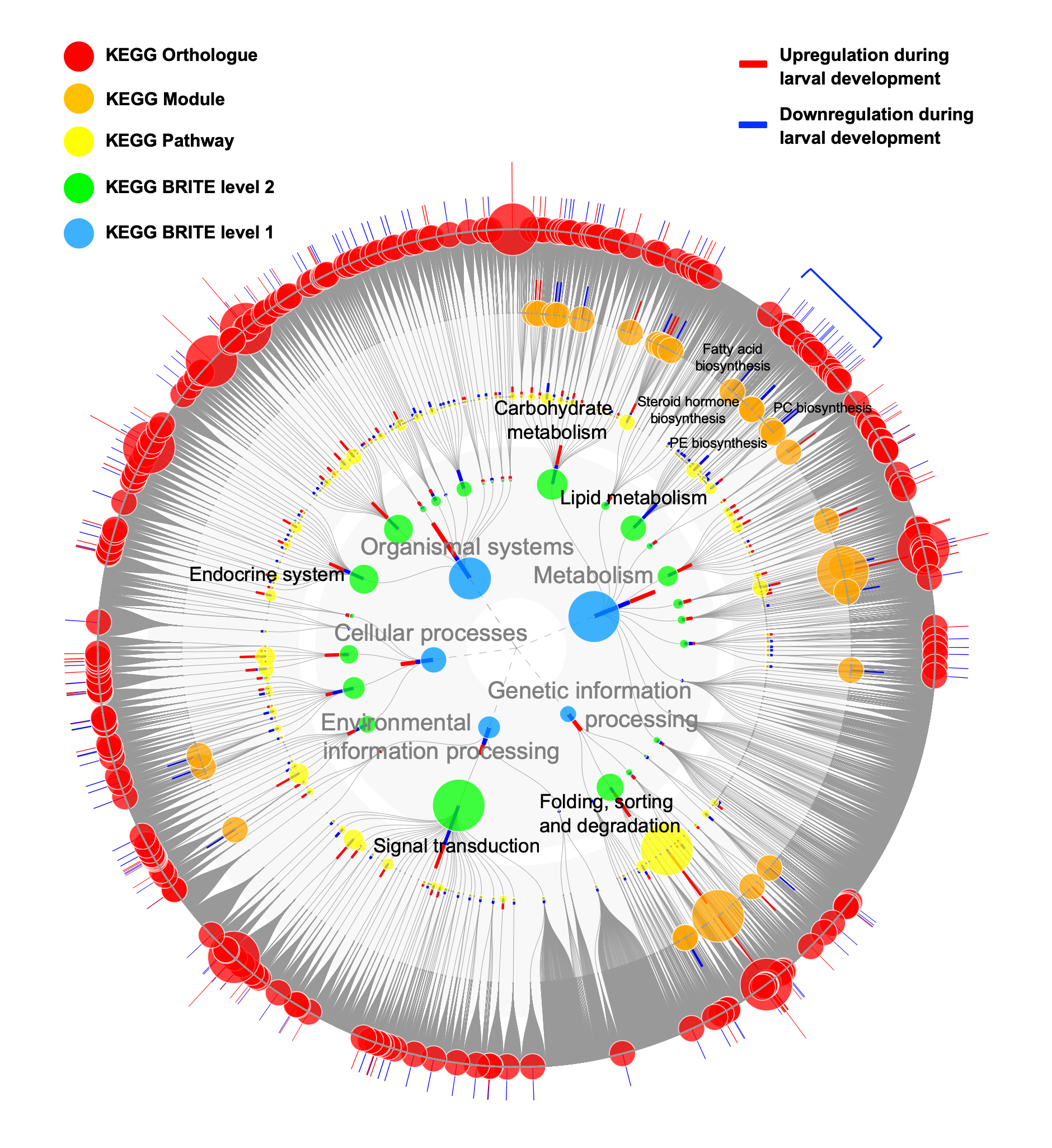

Supplement: S2 Fig — Functional annotation of mRNAs and proteins differentially transcribed/expressed between exsheathed L3s (xL3s) at 0 h and xL3s at 24 h. Annotation using the Kyoto Encyclopedia of Genes and Genomes (KEGG) database (employing Orthologue (red), Module (orange), Pathway (yellow) and BRITE levels 2 (green) and 1 (blue); see Materials and methods section). Significantly up-regulated (red) or down-regulated (blue) molecules and pathways are indicated. (TIF) [file ppat.1007960.s007.tif]
